# A Bilateral Symmetric Accessory Coracobrachialis Muscle Combined With an Interconnection of the Musculocutaneous Nerve With the Median Nerve

**DOI:** 10.7759/cureus.43496

**Published:** 2023-08-14

**Authors:** George Tsakotos, George Triantafyllou, Łukasz Olewnik, Georgi P Georgiev, Christos Koutserimpas, Vasileios Karampelias, Nicol Zielinska, Maria Piagkou

**Affiliations:** 1 Anatomy, National and Kapodistrian University of Athens, Athens, GRC; 2 Anatomical Dissection and Donation, Medical University of Lodz, Lodz, POL; 3 Orthopaedics and Traumatology, University Hospital Queen Giovanna - ISUL, Sofia, BGR; 4 Orthopaedics and Traumatology, 251 Hellenic Air Force General Hospital of Athens, Athens, GRC

**Keywords:** anatomical variation, anastomosis, median nerve, musculocutaneous nerve, interconnection, accessory head, coracobrachialis muscle

## Abstract

This report describes a bilateral symmetric accessory coracobrachialis muscle variant coexisting with a unilateral interconnection of the musculocutaneous nerve and the median nerve. An 80-year-old female cadaver was dissected. The bilateral coracobrachialis muscle variant consisted of three heads: two superficial heads and one deep head. One superficial head arose from the tip of the coracoid process, while the other originated from the short head tendon of the biceps brachii. The deep head of the coracobrachialis muscle emerged from the base of the coracoid process. The musculocutaneous nerve bilaterally coursed between the superficial and deep heads. On the right side, the three-headed coracobrachialis muscle coexisted with an ipsilateral interconnection of the musculocutaneous nerve and the median nerve, located at the lower third of the arm. While the presence of a unilateral three-headed coracobrachialis muscle is not rare (with a prevalence range of 0-22.2%), as well as the distal interconnection between the musculocutaneous nerve and the median nerve at the lower third of the arm (with a prevalence range of 1.8-53.6%), the coexistence of the current bilateral three-headed coracobrachialis muscle variant with the distal interconnection of the musculocutaneous and median nerves is quite unusual. A similar report underscores the finding of the bilateral coracobrachialis muscle variant.

## Introduction

The anterior arm compartment consists of the biceps brachii muscle (BB), the coracobrachialis muscle (CB), and the brachialis muscle (B). CB typically originates from the coracoid process (CP), along with the BB short head (BBsh), and inserts into the humeral shaft, above the middle of the humerus. Usually, the musculocutaneous nerve (MCN) penetrates CB [1,2] and innervates it. Atypically, CB receives aberrant innervation from fibers of the lateral cord (LC), and/or the medial cord (MC), and/or the posterior cord (PC), and/or the median nerve (MN) of the brachial plexus [1]. Although classic anatomy textbooks describe the CB of a single head as having the typical anatomy, many published cadaveric studies concluded to a CB with superficial and deep heads (SHs and DHs) [1]. Mori [3] recorded the CB division into SHs and DHs in 24%, contrary to the studies by El-Naggar [4], Ilayperuma et al. [5], Szewczyk et al. [6], and Piagkou et al. [7], which concluded to a higher frequency ranging from 42.6% to 94.4%. Ilayperuma et al. [5], Szewczyk et al. [6], and Piagkou et al. [7] recorded the MCN non-penetration into CB in an incidence ranging from 11.1% to 49.5% and correlated the MCN medial course with the one-headed CB. The MCN course was correlated with the number of CB heads, specifically when CB consisted of two heads or more, MCN coursed between CB heads [5,7]. MCN commonly gives off the communicating branch(es) to the MN, the so-called MCN and MN interconnection (IC) (MCN-MN), occurring from 1.8% to 53.6% [8,9]. The MCN-MN IC has been extensively studied and classified [9-11]. The current cadaveric report identified a bilateral (symmetric) three-headed CB and a unilateral MCN-MN distal IC. The embryological background of these variants and a thorough literature review are provided.

## Case presentation

**Figure 1:**
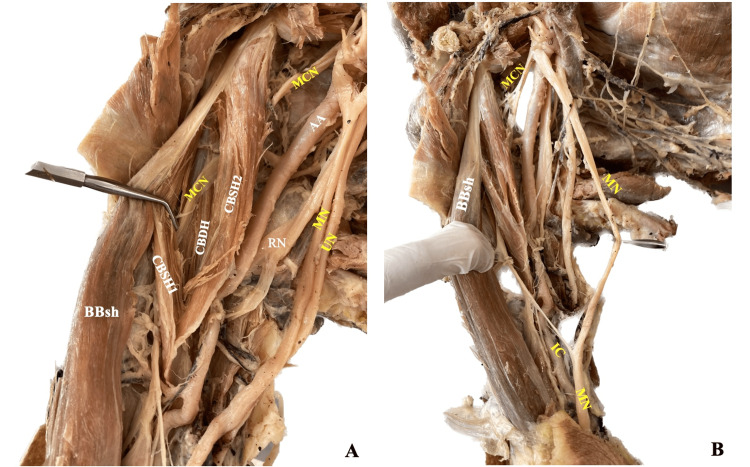
The right-sided three-headed coracobrachialis muscle (CB) variant (A) The three-headed coracobrachialis muscle (CB) with two superficial heads (CBSH1 and CBSH2) and one deep head (CBDH), and the musculocutaneous nerve (MCN) coursed through them. (B) The interconnection (IC) from the MCN to the median nerve (MN) at the lower third of the arm. BBsh: biceps brachii short head; UN: ulnar nerve; AA: axillary artery.

During routine dissection, an unusual bilateral and symmetric three-headed CB was identified, along with a right-side IC of the MCN with the MN. The dissection was performed on an 80-year-old formalin embalmed female cadaver of Greek origin, donated to our Anatomy Department, through the Body Donation Program after written informed consent. The skin, subcutaneous fat, and superficial fascia of the upper limb were dissected, and all muscles of the anterior and posterior arm compartments were exposed from their proximal to the distal attachment. The muscles were carefully examined for a typical or variant attachment, morphology, and innervation. Upper limbs were free of any physical deformity or trauma. The right-side three-headed CB is constituted by two SHs and one DH (Figure 1A). One SH of 147.2 mm in length, originated from the CP tip and fused with the origin of the BB short head. The other SH 119.1 mm in length arose distally from the BBsh. The DH 149.9 mm in length emanated from the CP base. The SHs fused and formed a common insertion with the DH at the middle third of the humeral shaft. The MCN coursed between SHs and DH. An IC of the MCN with the MN was observed 25.5 mm proximal to the cubital fossa (Figure 1B).

**Figure 2:**
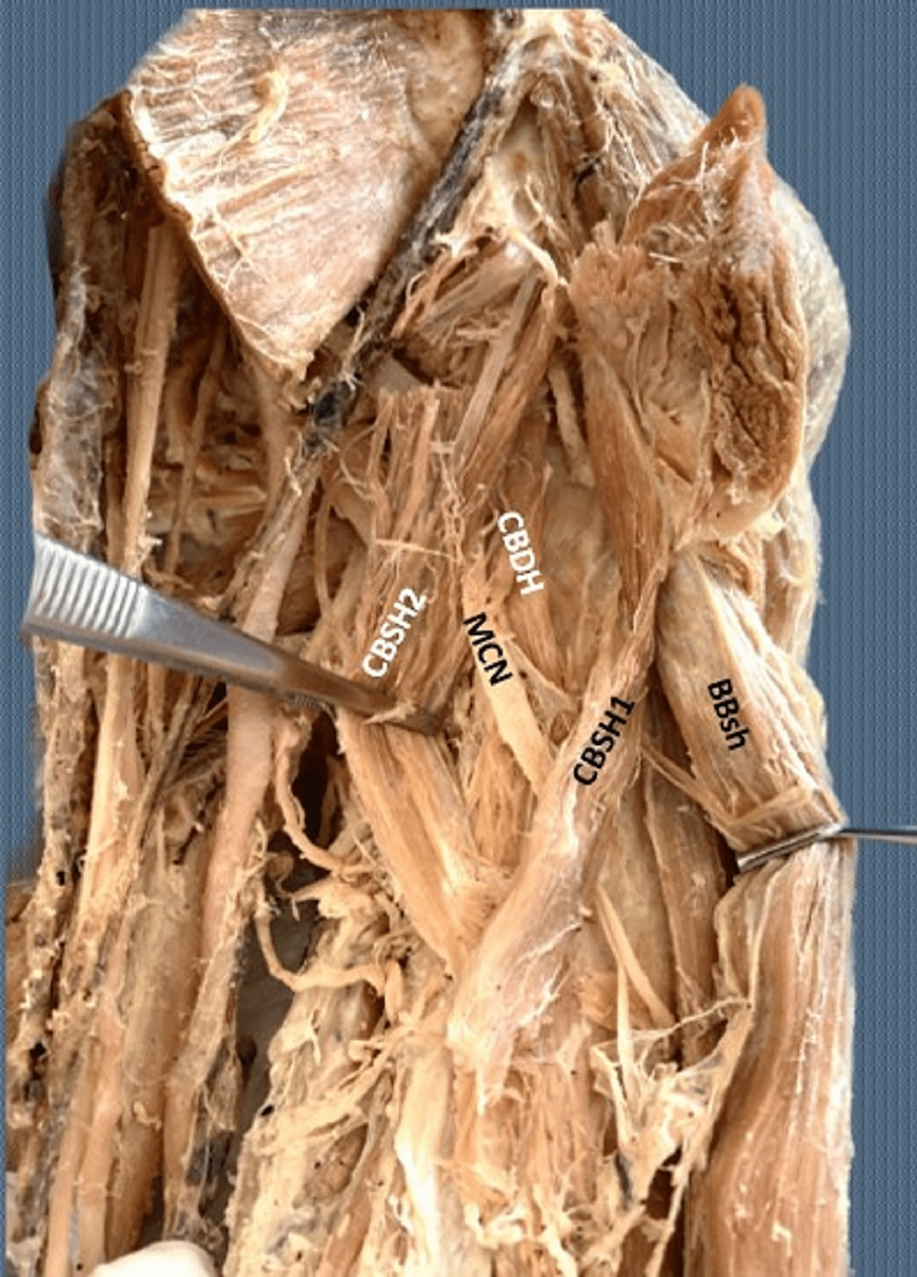
The left-sided three-headed coracobrachialis muscle (CB) variant The left-sided three-headed coracobrachialis muscle (CB) with two superficial heads (CBSH1 and CBSH2) and one deep (CBDH) head, with the musculocutaneous nerve (MCN) passing posterior to the superficial heads and anterior to the deep head. BBsh: biceps brachii short head.

The left-sided three-headed CB variant was symmetrical (Figure 2). One SH 159.2 mm in length originated from the CP tip, in common with the origin of the BBsh, and the other SH 115.1 mm in length arose from the BBsh tendon. The DH, 138.4 mm in length, originated from the CP base. The MCN passed between SHs and DH. No other variants were identified. 

## Discussion

In this report, a bilateral and symmetric three-headed CB was identified coexisting with a unilateral IC of MCN with the MN. CB variants have been systematically investigated in a few studies and were occasionally described in case reports (Table 1). El-Naggar [4] identified the CB typical form, consisting of two heads (one SH and one DH), with the MCN passing through them. In cases of one-headed CB, the MCN had a medial course in relation to the muscle. Ilayperuma et al. [5] identified a two-headed CB in 83.33% with the MCN perforating SH and DH, and a one-headed CB in 16.67% with an MCN of a medial course in relation to the CB. A statistically significant difference between the mean values of CB length, width, and thickness was recorded, those parameters were greater in males than in females [5]. Szewczyk et al. [6] proposed a classification for the CB morphological variants. CB type I variant was considered a single muscle originating from the CP, medially and posteriorly to the BBsh. Type II was a two-headed CB originating from the CP and tendon of the BBsh (type IIa) or both heads from the CP (type IIb). The three-headed CB (type III) had two heads originating from the CP and one head from the BBsh. Contrariwise to previous studies, Szewczyk et al. [6] detected the commonest variant, the one-headed CB (49.5%), followed by the two-headed (42.6%) and three-headed CB (7.9%). In almost all cases of two or more heads (98%), the MCN pierced CB except for one case of a two-headed CB that was not pierced by the MCN. In a separate cadaveric series, Piagkou et al. [7] identified the commonest form of a two-headed CB (62.97%).

**Table 1:** The cadaveric reports on the coracobrachialis muscle (CB) variant morphology (accessory heads) R: right; L: left; CP: coracoid process; BBsh: biceps brachii short head; TB: triceps brachii; MCN: musculocutaneous nerve; RA: radial artery; MN: median nerve; IC: interconnection; ND:  no data; MIS: medial intermuscular septum; CO: common origin.

Author(s)	Year	CB accessory heads (H)	Variant heads SH-superficial heads DH-deep heads AH-accessory head	Side	Attachments’ site		MCN relationship	Coexisted variants		
					Origin	Insertion		Muscular or osseous	Neural	Arterial
Catli et al. [12]	2012	3Hs	1st and 2nd	R	CP – 1st SH and 2nd DH	Middle humeral third	Between 2Hs	4H BB	MCN-MN IC	RA high origin
			3rd (capsular head)	R	Articular capsule of the glenohumeral joint	Middle humeral third				
Filippou et al. [13]	2023	6Hs	1st, 2nd, and 3rd (SHs)	R	CP tip	Middle humeral third shaft	Between SHs and DHs		MN atypical formation MN LR atypical course through CB heads	Variant AA branching pattern
			4th, 5th, and 6th (DHs)	R	BBsh tendon					
		5Hs	1st, 2nd, and 3rd (SHs)	L	CP and BBsh tendon	Middle humeral third	Between SHs and DHs		IC of the LR of the MN with the MCN	
			4th and 5th Hs (DHs)	L	CP base and shoulder capsule					
Georgiev et al. [14]	2017	3Hs	1st H	R	from the superior scapular notch	Upper third of the medial part of MIS	ND	ND		
			2nd H	R	CP tip					
			3rd H	R	CP (with the 2nd H)	Medial humeral epicondyle				
Gupta et al. [15]	2012	3Hs	AH (3rd head)	R	CP base and inferior surface	Medial border of humeral shaft	between SH and DH		CB accessory supply from LC of BP	
Kopuz et al. [16]	2003	3Hs	AH (3rd H)	R	CP and capsule of the shoulder joint	Fusion with the muscle belly	Between SH and DH	ND		
Olewnik et al. [17]	2021	CB superior muscle	Accessory muscle belly	R	CP (above CB origin)	CB common tendon	LC of BP passed between CB two bellies	ND		
Olewnik et al. [18]	2020	4Hs	1st and 2nd head	R	CP accessory apex	CB common tendon	MCN and MN passed between 1st and 2nd H	Split CP		
			3rd Head	R	CP apex (with BBsh)	CB common tendon				
			4th Head	R	CP inferior surface	Fusion to brachialis + CB common tendon				
Potu et al. [19]	2008	Variant insertion	Accessory slip	R	CB superficial fibers	Anteromedial aspect of the medial epicondyle	Piercing CB	ND		
Zielinska and Olewnik [20]	2022	6Hs	All six heads	R	Attachment on the medial surface of the humeral shaft	Proximal attachment with shBB	piercing CB 4th H	ND		

Piagkou et al. [7] identified the three-headed variant in 22.2%, with a bilateral appearance in the low prevalence of 6.67%. Similar unilateral variants were described by Kopuz et al. [16], Catli et al. [12], Gupta et al. [15], Georgiev et al. [14], and Zielinska et al. [21]. In Piagkou et al. [7] series, the four-headed CB variant had an incidence of 3.7% (1/27 upper limbs). Olewnik et al. [18] described a unilateral four-headed CB (accessory heads of different origins) in coexistence with a split CP with an accessory apex and a tunnel formation created from the CB two heads for the passage of both MCN and MN [18]. Zielinska and Olewnik [20] presented a unilateral six-headed CB, and Filippou et al. [13] identified a bilateral asymmetrical multiplication of the CB heads (a right-side six-headed CB in coexistence with a contralateral five-headed CB). 

The muscles of the anterior arm compartment arise from a common pre-muscular mass. They can be identified as separate structures in embryos 14-16 mm in length. The proximal end of the common mass differentiates earlier than the distal end. CB accessory heads probable are formed during the stage of differentiation in an embryo of 11-19 mm in length [22]. The nerves developed between the 4th and 7th embryonic week. Nerves supplying the limbs form a plexus by connecting loops between nerve fibers, hence any differentiation during this complex procedure could lead to an aberrant nerve supply and nerves’ ICs [23]. To understand the aberrant CB morphology, it is important to point out the CB phylogeny. Wood [24] identified in amphibians, reptiles, and monotremes the CB division into three distinct parts: (1) the CB brevis (CBB) or superior, (2) the CB medius (CBM), and (3) the CB longus (CBL). Typical CB in humans is formed of one muscular part, probably the CBM or the fusion of two heads (CBM and CBB) [25]. Hence, the aberrant muscles could be remnants that failed to disappear (CBL) or fuse to form a single muscle (CBM and CBB) [25].

The above-mentioned CB variants consisted of supernumerary heads. Wood [24, 26] described the CB variants as having aberrant origins and insertions. A CB variant is the CBL, which usually originated from the CP and variably is inserted into the humerus, and/or the fibrous band of the medial intermuscular septum (ligament of Struthers), and/or the medial supracondylar ridge, and/or the medial epicondyle, or an atypical supracondylar process [1]. The coracocapsularis muscle (of Wood) (1864), originated from the CP and was inserted into the shoulder capsule. Zielinska et al. [27] identified the CBL in 11% and proposed a classification for its variants. Georgiev et al. [14] described a novel CB variant, the “coracoepitrochlearis muscle,” consisting of three parts. The third part originated from the CP and was inserted into the medial humeral epicondyle [14]. The coracoepitrochlearis muscle differs from the CBL in its proximal and distal insertions. Georgiev et al. [28] also described an unreported CBL variant, the “humeroepitrochlearis muscle,” originating from the medial surface of the middle part of the humerus and inserted into the medial humeral epicondyle. CBB (of Cruveilher) [29] was recently identified by Olewnik et al. [17], originating proximally from the CP and distally below the lesser humeral tuberosity.

Overall, the prevalence of MCN variants is estimated at 20% [30]. In the current case, following the Le Minor classification [10], the interconnection (IC) branch from the MCN to the median nerve (MN) corresponds to type II variation. According to Venieratos and Anagnostopoulou [11] classification, the current case is classified as type II IC of the MCN with the MN distal to CB. IC between MN and MCN occurs in the lower third of the arm in 8%, similar to the current case [1]. The frequency of ICs between MCN and MN is much higher, while in the first large study, it was identified in 36% [31]. Guerri-Guttenberg and Ingolotti [9] identified one IC between MCN and MN in 53.6%, and only an IC of the MCN-MN was recorded distal to the point of the MCN to CB (7.7%), close to Tountas and Bergmann's [1] results. Interestingly, in Sirico et al. [30] meta-analysis, the most frequent region of the MCN variant (including the MCN-MN IC) was between the exit or underneath CB in 45.97%, in contrast to the studies of Guerri-Guttenberg and Ingolotti [9] and Tountas and Bergmann [1]. This difference could be justified by the fact that the subject of Sirico et al.'s [30] meta-analysis was focused in general on the MCN variants and not only on the ICs between MCN and MN. The variable incidence of the MCN-MN IC among different studies is summarized in Table 2.

**Table 2:** Incidence of the interconnection of the musculocutaneous nerve (MCN) with the median nerve (MN) among different studies with emphasis on the lower third of the arm, as in the present case ND: no data.

Author(s)	Year	Population	Sample (number of specimens)	Incidence (%)	Incidence at the arm lower third (%)
Kerr [32]	1918	American	75	24	ND
Venieratos and Anagnostopoulou [11]	1998	Greek	158	13.9	6.3
Olave et al. [33]	2000	Chilean	32	31.3	12.5
Choi et al. [34]	2002	British	276	19.2	10.1
Uysal et al. [35]	2003	Turkish	200	ND	1
Loukas and Aqueelah [36]	2005	Dutch & American	258	46	16.3
Chitra [37]	2007	Indian	50	26	8
Uysal et al. [38]	2009	Turkish	140	10	14.3
Guerri-Guttenberg and Inglotti [9]	2009	Argentinian	56	53.6	7.7
Kumar et al. [39]	2013	Indian	50	28	4
Caetano et al. [40]	2016	Brazilian	40	25	7.5
Claassen et al. [8]	2016	German	167	1.8	ND
Hayashi et al. [41]	2017	Japanese	130	23.8	3.1
Kara et al. [42]	2018	Turkish	50	4	0
Ghosh et al. [43]	2022	Indian	60	3.3	ND

Kosugi et al. [44] identified MCN-MN IC in coexistence with BB supernumerary heads in 54.7%. Hence, they supported that the supernumerary heads’ presence influences the MCN course and branching pattern. While muscles’ formation is completed before nerves’ formation, a developmental problem in muscle differentiation may lead to aberrant innervation. This theory was highlighted by Piagkou et al. [7], who identified the MCN-MN IC in coexistence with CB supernumerary heads in 11.1%.

The knowledge of possible variants, such as the three-headed CB, could prove useful since they are frequently accompanied by concomitant MCN variants. CP and CB represent a common site of surgical interventions, especially for shoulder surgeons. In recurrent or primary anterior shoulder dislocations with large glenoid deficits (>20%), the Latarjet procedure may be beneficial, which includes CP osteotomy and transfer of the osteotomized part along with the attached CB's tendons to the anterior part of the glenoid covering the deficit [45]. This can lead to MCN injury, which is a widely known complication in procedures around the anterior shoulder region; transient lesions of the MCN may also occur [46]. CB variants could also provoke subcoracoid impingement [47,48] and impede the modified Boytchev procedure for the treatment of anterior shoulder dislocation [49]. Potential injury to the MCN could be a significant intraoperative complication in these procedures. MCN surgical anatomy has been documented and studied in terms of the Latarjet operation due to its close anatomical relevance and serious injury-related complications [45,50]. Careful dissection and awareness of these cases are of utmost importance to avoid such adverse events. Other procedures, including CP internal fixation and acromioclavicular dislocation, also demand a careful approach to this anatomical area [7,51]. Moreover, the multiple CB heads in association with MCN variants may lead to entrapment syndromes in anatomical regions that are not normally involved [16]. Meticulous clinical examination and further investigation through magnetic resonance imaging (MRI), for the detection of the multiple heads and identification of the possible entrapment sites [48,52], as well as electromyography, may be crucial for adequate clinical decisions.

## Conclusions

CB supernumerary heads are not uncommon. However, the bilateral symmetrical CB multiplied variant (three and more heads) is quite unusual. Variants in muscle differentiation could lead to aberrant nerve formation, and thus the coexistence effect of muscular and nerve variants is of importance. The knowledge of the altered anatomy in the anterior arm compartment is paramount for orthopaedic surgeons to prevent iatrogenic injury.
